# Risk Factors Influencing Cyberbullying Perpetration among Middle School Students in Korea: Analysis Using the Zero-Inflated Negative Binomial Regression Model

**DOI:** 10.3390/ijerph18052224

**Published:** 2021-02-24

**Authors:** Kyung Im Kang, Kyonghwa Kang, Chanhee Kim

**Affiliations:** 1 Department of Nursing, College of Medicine, Dongguk University, Gyeongju 38066, Korea; fattokki@gmail.com; 2 Department of Nursing, Chungwoon University, Hongseong 32244, Korea; healerkang@gmail.com; 3 College of Nursing, Dong-A University, Busan 49201, Korea

**Keywords:** cyberbullying, middle school students, zero-inflated negative binomial model

## Abstract

This cross-sectional descriptive study identified risk factors and predictors related to the perpetration of and potential for cyberbullying among adolescents, respectively. The analysis included a zero-inflated negative binomial regression model. Data were assessed from 2590 middle-school student panels obtained during the first wave of the Korean Child and Youth Panel Survey 2018. Of these respondents, 63.7% said they had not experienced the perpetration of cyberbullying. However, a subsequent count model analysis showed that several factors were significantly associated with cyberbullying, including offline delinquency, aggression, smartphone dependency, and smartphone usage on weekends (either 1–3 h or over 3 h). A logit model analysis also showed several predictive factors that increased the likelihood of cyberbullying, including gender (boys), offline delinquency, aggression, smartphone usage during weekdays (1–3 h), computer usage during weekends (1–3 h), and negative parenting. These identified risks and predictors should be useful for interventions designed to prevent the perpetration of cyberbullying among middle school students.

## 1. Introduction

The increasing popularity of internet-enabled devices (e.g., smartphones) and electronic communication tools (e.g., social networking services) has substantially influenced peer interactions among adolescents in both the offline and online social contexts [1,2,3]. Since COVID-19 has drastically reduced the rate of school attendance and ability for these individuals to engage in close proximity, most traditional education methods have been replaced by the online learning format. In this environment, most instances of peer communication are also being conducted via digital services and devices, including e-mail, mobile phones, and social networking services (SNS). Notably, a recent survey study reported that excessive Internet usage is increasing among adolescents in South Korea [4]; the more time they spend on digital services, the more likely they are to be exposed to cyberbullying, both nationally and internationally [5,6]. 

Bullying is defined as a form of aggressive behavior, including the intent to harm interpersonal relationships; it is also characterized by an imbalance of power [7]. Traditional school bullying has expanded beyond the school environment and into the cyber realm, where cyberbullying is considered part of a relational context involving the perpetration of bullying and victimization [3,8,9]. Many adolescents are evolving these issues into more diverse and complex forms, including overlapping verbal and emotional harassment both online and offline by ostracizing specific targets in the cyber realm [5,8,10,11]. A nationwide survey of cyberbullying conducted by the Korean Institute of Criminology [5] found that 51% of adolescents had experienced cyberbullying. Even when considering the different measurement methods, this is very high when compared to the rates found in other countries. For instance, studies have shown figures of 21.0% in the United States [10], 27% in Germany [12], and 40.6% in Australia [2]. The rate in Korea is also much higher than the 37% reported as a worldwide average [13]. 

Cyberbullying refers to any continuous and repeated psychological attacks via information and communication devices and/or the dissemination of personal or false information related to specific youths, thus causing the affected person to feel pain [6,14,15,16]. Cyberbullying places further emphasis on the characteristics of online services (e.g., anonymity, non-face-to-face interactions, rapid proliferation, and sharing) and the spatiotemporal transcendence of online social spaces [1,14,16]. Cyberbullying perpetration begins with personal and situational factors that activate hostile thoughts while increasing arousal [14]. When individuals evaluate an aggressive response to a specific person as appropriate, they immediately use a digital device to engage in aggressive behavior (e.g., sending vulgar text messages). Cyberbullying perpetrators also encourage others to participate. This is crucial, as cyberbullying then intensifies a variety of problems known to affect adolescents, including issues related to social adaptation (e.g., internalization and externalization) and the rate of cyberbullying victimization itself; it also results in serious health disruptions via physical threats and psychological deterioration [17]. Involvement in cyberbullying perpetration not only damages health-related quality of life, but also increases the risk of victim suicide [14,16,18,19,20], which has emerged as a very serious public health problem throughout the world. 

Systematic reviews on the factors that affect cyberbullying perpetration [6,21] have identified self-esteem, empathy, aggression, depression, social withdrawal, the frequency of Information and Communications Technology (ICT) usage, academic achievement, parental interaction or monitoring, peer influence, school commitment, and school climate, which is further divided into categories of person-related, media-related, and environmental (family, peers, and school). However, most of the findings included in this study’s investigation [6] were identified via linear regression models, such as the logistic regression [2,12]. As neither the health-related nor environmental impacts of COVID-19 have been eliminated, digital service usage will continue to increase among adolescents, which is expected to further aggravate existing negative aspects. This makes it important to accurately identify the factors that affect cyberbullying perpetration, especially in the interventional context, as there are still debates on which factors should be targeted [6,22]. In this case, continued efforts are needed to reduce the related problems through a better understanding of the risks associated with cyberbullying, especially given the growing trend of perpetration.

The distributions of risk behaviors such as cyberbullying will contain a large number of zero values. Although there are severe consequences to cyberbullying perpetration behaviors, many adolescents report that they have not experienced any such instances. Such zeros in the frequency create inaccurate results when conventional normalization statistical methods are applied. There are particular concerns about the linear regression model, specifically regarding both the overdispersion of count data (negative binomial) and unobserved heterogeneity due to excess zeros [16,23,24]. However, these concerns can be addressed via the zero-inflated negative binomial (ZINB) model. When attempting to identify the risk factors for cyberbullying among adolescents, the ZINB model can handle excessive zeros with over-dispersion (negative binomial) in the observed data and is associated with one important distinction in how the zero counts are interpreted and analyzed.

Based on the above, this study identified risk factors and predictors related to the perpetration of and potential for cyberbullying among adolescents via ZINB regression analysis.

## 2. Materials and Methods

### 2.1. Study Participants and Procedures

The data investigated in this study were obtained during the first wave of the Korean Child and Youth Panel Survey 2018 (KCYPS 2018), which was conducted using a longitudinal panel design via the National Youth Policy Institute (NYPI) in Korea. Study participants were selected through the stratified multi-stage cluster sampling method. A total of 16 administrative districts were stratified; schools were then randomly chosen from each district via proportionate probability sampling in accordance with the population rate. Finally, one class was randomly chosen from each school. The first wave of the KCYPS 2018 consisted of fourth-grade elementary school student panels and first-grade middle school student panels. The first wave of data collection was conducted from August to November 2018 after obtaining IRB approval from the IRB committee of NYPI [25]. The explanatory documents were distributed and informed consent for participating in this survey was acquired from the legal guardians of adolescents [25]. In addition, the survey was conducted only by visiting schools that agreed to the survey and was done only when the legal guardians of the adolescents agreed to participate in the survey [25]. This study ultimately analyzed data from 2590 first-grade middle school student panels with a mean age of 13 years.

### 2.2. Measurements

The frequency of cyberbullying perpetration was measured based on participant answers to the 15 items shown in Table 1. They were asked how frequently (if at all) they had experienced involvement in the perpetration of cyberbullying over the 12-month period prior to investigation; this was rated using a six-point scale, in which “never” = 0, “one to two times yearly” = 1, “once per month” = 2, “two to three times per month” = 3, “once weekly” = 4 and “many times weekly” = 5. Here, higher scores indicated more frequent experiences with the perpetration of cyberbullying (Cronbach’s alpha of 0.56).

Participants measured a total of 15 experiences related to their involvement in offline delinquency over the 12-month period prior to investigation, including smoking, drinking alcohol, unexcused absences, running away from home, excessive teasing or mocking of others, out-casting others, gang fights, severely beating others, threatening others, taking money or possessions from others, stealing money or possessions, engaging in sexual relations, sexual abuse or harassment, gambling for money, and verbal abuse (or foul language). Each item was rated using a six-point scale, in which “never” = 0, “one to two times yearly” = 1, “once per month” = 2, “two to three times per month” = 3, “once weekly” = 4 and “many times weekly” = 5. Here, higher scores indicated more frequent experiences with offline delinquency (Cronbach’s alpha of 0.66).

Aggression was assessed using a six-item scale developed through previous research [26]. This scale measures not only the aggression that an individual feels inside, but also the parts expressed to the others such as peers or family members. Each item was rated using a four-point Likert scale, in which 1 = “not at all” and 4 = “very strongly.” Here, higher scores indicated higher levels of aggression (Cronbach’s alpha of 0.84 in this study). 

Depressive symptoms were assessed using 10 items from the symptom checklist-90-Revised [27]. Each item was rated using a 4-point Likert scale ranging from 1 = “not at all” to 4 = “very strongly.” All items were reverse-coded, with higher scores thus indicating higher levels of depression (Cronbach’s alpha of 0.92 in this study). 

Social withdrawal was assessed using a revised version of the Behavior Problem Scale for Children and Adolescence [28]. More specifically, the scale consisted of five items that were rated using a 4-point Likert scale ranging from 1 = “strongly disagree” to 4 = “strongly agree,” with higher scores indicating increased levels of social withdrawal (Cronbach’s alpha of 0.87 in this study).

The Rosenberg Self Esteem Scale [29] was used to measure self-esteem. The scale consists of 10 items that are rated using a four-point Likert scale ranging from 1 = “very true for me” and 4 = “not at all true for me.” Negative items were reverse coded, so that higher scores indicated higher self-esteem (Cronbach’s alpha of 0.87 in this study). 

Smartphone dependency was assessed using the Smartphone Addiction Proneness Scale (K-SAS) [30], which was developed by the National Information Society Agency (NIA) in Korea. The K-SAS consists of 15 items across the six subdomains of daily life disturbance, withdrawal, tolerance, and virtual world orientation. All items were rated using a four-point Likert scale ranging from 1 = “not at all” to 4 = “very strongly”. Positive items were reverse coded, so that higher scores indicated increased smartphone dependency (Cronbach’s alpha of 0.88 in this study).

The amount of technology usage was measured to determine how often participants used smartphones and computers during both the weekdays and weekends. The amounts reported by the adolescents were coded as follows: 0 = “less than 1 h”, 1 = “1–3 h”, 2 = “over 3 h”.

The Korean Version of the Parents as Social Context Questionnaire for Adolescents (K-PSCQ) [31] was used to measure perceived positive and negative parenting [32]. The K-PSCQ measures the three positive dimensions of structure, warmth, and autonomy support, and the three negative parenting dimensions of coercion, rejection, and chaos. All items were rated on a four-point Likert scale ranging from 1 = “very true for me” and 4 = “not at all true for me”, with higher scores indicating increased levels of positive and negative parenting, respective to the dimensional components (Cronbach’s alphas for positive and negative parenting of 0.92 and 0.87, respectively). 

### 2.3. Data Analysis

**Figure 1:**
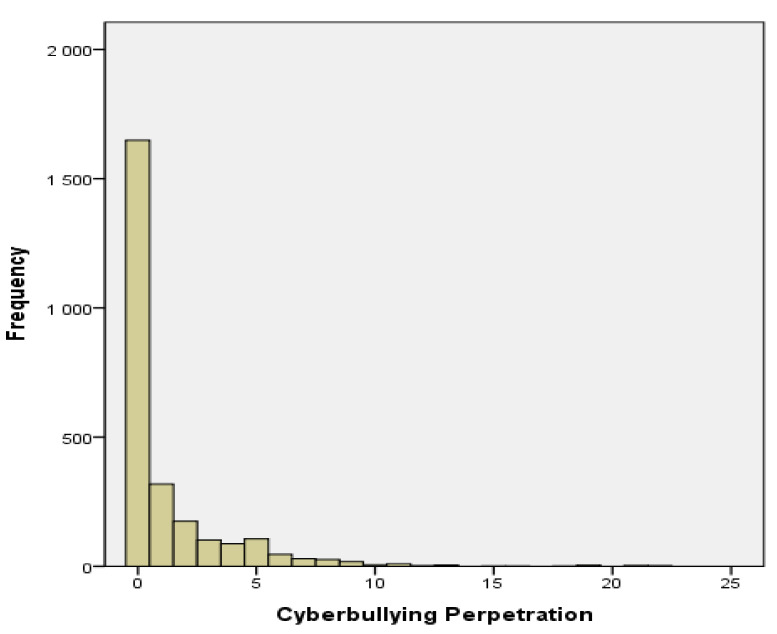
Frequency of cyberbullying perpetration among South Korean middle school students.

Data analysis was conducted using the SPSS version 22.0 software (IBM Corp., Armonk, NY, USA) and Stata 15.1 program (Stata Corp., College Station, TX, USA). First, descriptive statistics were employed. The outcome variable was set as the frequency of cyberbullying perpetration and contained nonnegative integer values. With respect to this variable, a total of 1649 (63.7%) adolescents answered “0” (Figure 1). Here, we used the ZINB regression model to examine which factors predicted the frequency of cyberbullying perpetration, particularly as it presents overdispersions and can adequately handle issues related to the presence of many zero values. Within the ZINB model, the large quantity of zero answers was assumed to be associated with a mixture of two discrete groups: the first was “true/structural zero,” or individuals who always report zero experiences of cyberbullying perpetration, while the second was “sampling zero,” or individuals who sometimes experience the issue, but had not done so within one year of the study period. As shown in previous research, the ZINB model can improve overall explanatory power by accounting for zero values [23,24]. The model estimation was undertaken with two latent groups: (1) the “0” group, with zero answers handled using a mixed strategy for the logit model, and (2) the group of respondents who reported at least a “1,” with answers analyzed using the count model [23,24]. In this context, the ZINB model was used to examine the logarithms of the logit model (which predicted the likelihood of future behavioral problems related to cyberbullying perpetration) and count model (which predicted the factors that influenced the severity of existing problems related to cyberbullying perpetration).

## 3. Results

### 3.1. Demographic Characteristics of the Study Participants 

As shown in Table 2, there were 2590 total adolescents (54.2% boys with a mean age of 13.00 years; Standard Deviation [*SD*] = 0.13). Among these participants, 76.3% perceived their economic status as medium. Table 2 also shows the means and standard deviations for the study variables.

### 3.2. ZINB Model Results for the Perpetration of Cyberbullying

Figure 1 shows the frequency of cyberbullying perpetration among participants. There was an excessive amount of zero values (63.7%), while the Vuong statistic was z = 8.49 and *p* > z = 0.000, thus confirming model suitability because the overdispersion issue was dissolved. Table 3 shows the ZINB model results. The count model analysis showed that high levels of offline delinquency (β = 0.089, *p* < 0.001), aggression (β = 0.050, *p* < 0.001), and smartphone dependency (β = 0.014, *p* = 0.013) were associated with increased cyberbullying behaviors. Participants who used smartphones between both 1–3 h (β = 0.669, *p* < 0.001) and over 3 h (β = 0.673, *p* < 0.001) during weekends also showed higher levels of cyberbullying behaviors when compared to those who used smartphones less than 1 h during weekdays. Meanwhile, the logit model analysis showed that participants who were boys (β = −0.330, *p* = 0.049) and had higher levels of offline delinquency (β = −1.371, *p* < 0.001), aggression (β = −0.109, *p* < 0.001), and perceived negative parenting (β = −0.029, *p* = 0.046) exhibited an increased likelihood of cyberbullying perpetration. Middle-school students who used smartphones between 1 and 3 h during weekdays (β = −0.643, *p* = 0.002) also showed a higher likelihood of cyberbullying perpetration when compared to those who used smartphones less than 1 h during weekdays. Finally, participants who used computers between 1 and 3 h during weekends (β = −0.430, *p* = 0.038) exhibited a higher likelihood of cyberbullying perpetration when compared to those who used computers less than 1 h during weekends.

## 4. Discussion

This study identified which factors affected the frequency and probability of cyberbullying perpetration among South Korean adolescents via the ZINB model. Here, the goal was to provide useful information for interventions aimed at the reduction and prevention of cyberbullying through various approaches based on relevant factors. Results showed that offline delinquency, aggression, and smartphone usage during weekdays or weekends (1–3 h) were the main common factors affecting both the increased frequency and probability of cyberbullying perpetration. These findings also support those of previous studies, both domestically and abroad, thus indicating that the above three factors should be targeted in relevant prevention and reduction measures. A variety of recent studies have consistently shown that several factors are significantly associated with cyberbullying, including offline delinquency, unauthorized school absences, antisocial behaviors, aggression against others (including peers) [33,34], and increased Internet usage and/or smartphone interaction hours [35]. In particular, Guo [33] found that offline delinquency was the strongest predictor for both cyberbullying and problematic behavior, thus showing that physical bullying often extends to cyberspace. Moreover, Gradinger and Strohmeier [34] identified that individuals who engaged in cyberbullying and/or offline delinquency commonly experienced difficulty when attempting to externalize their adjustment problems. This emphasizes the need to not only reduce bullying itself, but also to help adolescents solve their adjustment problems through healthy externalization. In sum, a combination of in-school counseling, personal and group programs, and educational measures are needed to prevent physical bullying and aggressive tendencies from transferring to the digital world; this should also help achieve other emotional and behavioral improvements. Regarding excessive smartphone usage, most schools now apply relatively strict regulations and controls, although the issue is already prevalent among adolescents in Korea [2]. Considering the fact that smartphone usage is more frequent in after-school academies and the home, increased parental guidance and monitoring should also be emphasized. Further, recent changes resulting from the COVID-19 pandemic have made it increasingly important to provide special guidance for adolescents who are active in the online-based education environment. This can be accomplished with the help of a variety of tools such as applications or functions that allow parents to remotely measure smartphone usage or tracking devices, including those that facilitate better communication and mediation.

The count model verified that smartphone dependency could also intensify the frequency of cyberbullying perpetration. This supports the findings of previous studies conducted among university students and adolescents [36,37]. Indeed, smartphones constitute a major medium for cyberbullying, with severe aggressive and/or delinquent behaviors seen in adolescents who are at risk for smartphone addiction [38]. In light of these characteristics, smartphone dependency likely intensifies cyberbullying. Moreover, both cyber victimization and cyberbullying tend to increase alongside the increased use of social network services (SNS), texting, and the Internet via smartphone devices, all of which may reach particularly severe levels during adolescence [39]. A domestic survey showed that 34.7% of middle school students in Korea fell into the risk group for smartphone dependency while also showing the highest vulnerability rate [40]. Further, mental health problems caused by smartphone dependency during middle school years may continue into later adolescence [41]. In this case, it is necessary to reduce the perpetration of cyberbullying through active efforts implemented by both schools and families, particularly in order to reduce and prevent smartphone dependency based on an accurate understanding of the problem.

Meanwhile, factors such as gender (male), the degree of computer usage on weekends, and negative parenting styles have also been found to increase the probability of cyberbullying perpetration. Notably, however, there are mixed results on how gender affects cyberbullying. Congruent with the results of this study, some investigations have shown that adolescent boys have an increased risk of cyberbullying perpetration [33,35,42,43], while others have indicated that gender has no significant effect in the adolescent context [36,44]. Meanwhile, Wright [45] interpreted that the phenomenon of cyberbullying was largely influenced by masculine tendencies among adolescents rather than physical gender itself. Basically, there is insufficient evidence for making conclusions about the relevant dynamics at this time. This means that additional studies and relevant interventions are needed to target the specific factors and dynamics of cyberbullying, particularly in terms of gender-based differences.

On the other hand, previous studies have shown a significant correlation between the amount of computer usage and the probability of cyberbullying among adolescents [2,46]. Specifically, adolescents may spend much of their spare time on computers in order to search for online information, play games, and engage in social networking [2,14,47]. Increased hours spent on the computer have been found to increase the probability of cyberbullying, especially when combined with problems such as the acquisition of harmful information, defects in ethical and moral values, depression and anxiety, and the lack of empathy [22,46]. For adolescents in the so-called digital generation, the ability to use information and communication technology (ICT) through various devices entails many advantages, and now constitutes a firm trend. The potential for Internet usage through computers to become another medium for cyberbullying [14] emphasizes the need for educational programs and interventions aimed at increasing self-control, the ability to recognize harmful factors, and the practice of healthy and effective computer usage. These efforts should play significant roles in the prevention of cyberbullying. 

Previous studies with results similar to those of this study have shown that negative parenting (especially authoritative and overly controlling attitudes or overprotection, low-quality parent-child relationships, and the inconsistent application of rules) is associated with increased cyberbullying [48,49]. On the other hand, research has also shown that emotional family support and proper control in the home are major factors for reducing the probability of cyberbullying [22,39]. Specifically, Mesch [50] suggested that parents could use two types of mediation to prevent cyberbullying, including (1) evaluative mediation (e.g., assessing the dangers of Internet use with children) and (2) restrictive mediation (e.g., setting appropriate limits). However, some studies have shown an insignificant correlation between parental restrictions/control and cyberbullying perpetration [51], in which case additional qualitative and quantitative studies are needed to achieve a more precise and profound understanding of these relationships and dynamics. As negative parenting is known to increase the probability of future cyberbullying perpetration among adolescents, various approaches and more careful considerations are also needed. This includes educational programs for parents, positive support and care for children, and family support through active communication.

Aside from the factors identified in this study, cyberbullying is also associated with multi-dimensional problems, such as juvenile offender individuality, values, motives, stress, anxiety, social awkwardness, the level of ICT usage, and lacking moral values [35,52]. Moreover, these factors may disrupt healthy mental and social development for adolescents [42]. In this regard, positive growth and development should be promoted based on an accurate consideration of the characteristics associated with known adolescent developmental stages and the various factors that are directly related to cyberbullying. These problems must be addressed through continual research, professional cooperation, and systematic practices designed to develop effective interventional approaches.

## 5. Limitations and Implications

This study’s results were generalized through an analysis of data collected by the Korean Children and Youth Panel Survey 2018, which is a nationally recognized source. It is meaningful in the sense that it applied the ZINB regression method to solve the problems of overdispersion in the frequency data of cyberbullying perpetration among middle school students and the heterogeneity of zero-inflated values. Indeed, the ZINB regression method was effective for identifying relevant factors that intensified the frequency of cyberbullying perpetration. It also enabled a broad understanding of the major factors that increase the likelihood of cyberbullying perpetration, specifically by verifying the predictors, which previous studies were unable to verify through the logistic regression method.

Despite its significant findings, this study also had some limitations. First, the influences of variables not included in the analysis cannot be completely excluded, particularly since this study utilized secondary data collected through a panel survey. Future studies should, therefore, investigate various other factors that can affect cyberbullying perpetration, including mental health issues experienced by adolescents. It is also important to identify any interactions between factors. Second, it is possible that adolescents responded with more socially desirable answers and/or did not answer questions related to sensitive topics (e.g., cyberbullying, delinquent behaviors, aggression, and parenting behaviors), and may have also felt pressure due to the large overall number of questions presented in the survey. As these factors may have partially influenced the low Cronbach’s alpha (0.56) value of the cyberbullying instrument, they should be considered when interpreting the data and results. Further, this means that additional studies are needed to investigate adolescent experiences with cyberbullying perpetration as well as any relevant details more accurately and in a more comfortable and secure environment. More effective help may thus be provided based on the results. Finally, the data used in this study were obtained through a cross-sectional survey, which limits the ability to estimate causality between factors. Future longitudinal studies may therefore provide more meaningful and practical information. In this regard, it is crucial to identify changes in the relationship between progress and the influencing factors of cyberbullying perpetration among adolescents. 

## 6. Conclusions

This study examined various factors related to cyberbullying perpetration among middle school students in Korea from different perspectives based on data from the Korean Children and Youth Panel Survey 2018. Throughout this process, we focused on factors that were likely increase the probability of cyberbullying perpetration among subjects without relevant experiences as well as the influencing factors relevant to individuals who had already experienced the problem. This study’s results constitute meaningful basic data for the development of policies and interventions aimed at reducing and preventing the many harmful outcomes of cyberbullying, which is a major side effect derived through unique characteristics of the digital generation. This is especially important for adolescents who partake in the many advantages provided by ICT, and who will, therefore, experience more online-based education and interaction due to environmental changes resulting from the COVID-19 pandemic. In terms of future research, the literature would benefit from comparative analyses involving relevant data from cyber aggressors and cyber victims in the adolescent context. Studies should also examine whether the views of adolescents are similar to those held by adults when considering political aspects and/or approaches to cyberbullying prevention.

## Figures and Tables

**Table 1:** Items related to the perpetration of cyberbullying.

Item No.	Content
1	I have sent abusive or harsh words to another person.
2	I have spread bad rumors about a person to others.
3	I have stalked another person by sending messages, photos, etc. against that person’s will.
4	I have sent or secretly delivered another person’s photos, bizarre pictures, images, or videos to others against that person’s will.
5	I have made fake accounts by stealing another person’s ID, then portrayed myself as that person in cyberspace.
6	I have doxed another person by posting their personal information (e.g., name, age, school, phone number) on the Internet.
7	I have extorted game money, game items, and cyber money.
8	I have made another person do “Wi-Fi shuttles” or “hot spot shuttles” (i.e., forced them to provide cellphone data for free).
9	I have sent sexual messages, obscene photos, or videos against the recipient’s will.
10	I have stopped others from leaving Internet chatrooms or repeatedly invited others against their will.
11	I have led another person to curse first or made that person appear to have personality issues by deliberately starting a quarrel.
12	I have used my smartphone to make another person do things against their will or force them to run (cigarette) errands.
13	I have made public online posts to attack another person.
14	I have made intensive attacks on another person in cyberspace.
15	I have intentionally refused to invite someone to a chatroom or ignored their comments or messages.

**Table 2:** Descriptive statistics for the general participant characteristics and study variables (*N* = 2590).

		*N* (%)	Mean (*SD*)	Range
Age			13.00 (0.13)	12–14
Gender Female		1185 (45.8%)		
Male		1405 (54.2%)		
Perceived Economic Status				
Low		349 (13.4%)		
Medium		1972 (76.3%)		
High		264 (10.2%)		
Cyberbullying Perpetration			1.23 (2.46)	0–22
Offline Delinquency			0.74 (2.24)	0–38
Aggression			11.50 (3.54)	6–24
Depression			17.99 (6.38)	10–40
Social Withdrawal			10.76 (3.75)	5–20
Self-Esteem			29.94 (5.04)	11–40
Smartphone Dependency			30.59 (7.32)	15–60
Technology usage	Smartphone Usage on Weekdays			
	Less than 1 h	642 (24.8%)		
	1–3 h	1409 (54.4%)		
	Over 3 h	539 (20.8%)		
	Smartphone Usage on Weekends	438 (16.9%)		
	1–3 h	1112 (42.9%)		
	Over 3 h	1040 (40.2%)		
	Computer Usage on Weekdays			
	Less than 1 h	1935 (74.7%)		
	1–3 h	538 (20.8%)		
	Over 3 h	117 (4.5%)		
	Computer Usage on Weekends			
	Less than 1 h	1497 (57.8%)		
	1–3 h	723 (27.9%)		
	Over 3 h	370 (14.3%)		
Positive Parenting			39.11 (5.38)	12–48
Negative Parenting			23.96 (6.29)	12–48
Relationship with Friends			40.63 (5.62)	15–52
School Satisfaction				
Not satisfied		360 (14.6%)		
Moderate		952 (38.5%)		
Satisfied		1159 (46.9%)		
Perceived Academic Achievement				
Low		174 (13.0%)		
Medium		497 (37.2%)		
High		666 (49.8%)		

*SD* = standard deviation.

**Table 3:** Zero-inflated negative binomial regression results for the frequency of cyberbullying perpetration (*N* = 2590).

		Count Model					Logit Model				
		β	*SE*	*z*	*p* > |*z*|	95% CI	β	*SE*	*z*	*p* > |*z*|	95% CI
Gender (ref. female)											
Male		−0.006	0.092	−0.07	0.944	−0.186, 0.173	−0.330	0.168	−1.97	**0.049**	−0.660, −0.001
Perceived Economic Status (ref. low)											
Medium		0.046	0.102	0.45	0.650	0.154, 0.247	−0.198	0.208	−0.96	0.339	−0.605, 0.208
High		0.043	0.149	0.29	0.772	−0.249, 0.335	0.303	0.297	−1.02	0.307	−0.885, 0.279
Offline Delinquency		0.089	0.011	8.31	**<0.001**	0.068, 0.110	−1.371	0.208	−5.06	**<0.001**	−1.902, −0.840
Aggression		0.050	0.012	3.97	**<0.001**	0.025, 0.074	−0.109	0.026	−4.24	**<0.001**	−0.159, −0.058
Depression		0.002	0.009	0.26	0.798	−0.016, 0.020	−0.029	0.018	−1.57	0.117	−0.065, 0.007
Social Withdrawal		−0.016	0.011	−1.52	0.129	−0.038, 0.005	0.029	0.023	1.29	0.196	−0.0151, 0.074
Self-Esteem		−0.016	0.010	−1.48	0.138	−0.036, 0.005	−0.015	0.021	−0.72	0.471	−0.056, 0.026
Smartphone Dependency		0.014	0.006	2.48	**0.013**	0.003, 0.025	−0.019	0.011	−1.66	0.096	−0.041, 0.003
Technology usage	Smartphone Usage on Weekdays (ref. less than 1 h)										
	1–3 h	−0.025	0.120	−0.21	0.83	−0.261, 0.211	−0.643	0.211	−3.04	**0.002**	−1.057, −0.229
	Over 3 h	0.105	0.145	0.72	0.470	−0.179, 0.0389	−0.354	0.270	−1.31	0.191	−0.883, 0.176
	Smartphone Usage on Weekends (ref. less than 1 h)										
	1–3 h	0.669	0.146	4.57	**<0.001**	0.382, 0.955	0.589	0.274	2.16	0.320	0.051, 1.124
	Over 3 h	0.673	0.153	4.40	**<0.001**	0.373, 0.974	0.278	0.296	0.94	0.348	−0.303, 0.859
	Computer Usage on Weekdays (ref. less than 1 h)										
	1–3 h	0.031	0.099	0.31	0.757	−0.163, 0.225	0.083	0.220	0.38	0.706	−0.349, 0.515
	Over 3 h	0.129	0.169	0.76	0.446	−0.203, 0.461	0.439	0.374	1.17	0.240	−0.294, 1.171
	Computer Usage on Weekends (ref. less than 1 h)										
	1–3 h	−0.136	0.101	−1.35	0.178	−0.334, 0.062	−0.430	0.207	−2.08	**0.038**	−0.836, −0.024
	Over 3 h	0.121	0.119	1.02	0.309	−0.112, 0.355	−0.379	0.277	−1.37	0.171	−0.921, 0.163
Positive Parenting		0.006	0.007	0.90	0.368	−0.007, 0.020	−0.004	0.015	−0.28	0.776	−0.034, 0.025
Negative Parenting		0.001	0.007	0.21	0.835	−0.012, 0.015	−0.029	0.015	−2.00	**0.046**	−0.058, −0.001
Relationship with Friends		0.007	0.007	0.91	0.362	−0.008, 0.021	−0.024	0.015	−1.64	0.101	−0.054, 0.005
School Satisfaction (ref. not satisfied)											
	Moderate	−0.133	0.160	−0.83	0.407	−0.448, 0.182	−0.182	0.351	−0.52	0.604	−0.870, 0.506
	Satisfied	−0.158	0.150	−1.05	0.293	−0.453, 0.137	−0.388	0.335	−1.16	0.246	−1.044, 0.268
Perceived Academic Achievement (ref. low)											
	Medium	0.050	0.106	0.48	0.634	−0.157, 0.258	0.196	0.224	0.87	0.382	−0.244, 0.636
	High	0.184	0.106	1.73	0.084	−0.025, 0.392	0.090	0.226	0.040	0.690	−0.353, 0.533

Bold values were significant.
